# The first metazoa living in permanently anoxic conditions

**DOI:** 10.1186/1741-7007-8-30

**Published:** 2010-04-06

**Authors:** Roberto Danovaro, Antonio Dell'Anno, Antonio Pusceddu, Cristina Gambi, Iben Heiner, Reinhardt Møbjerg Kristensen

**Affiliations:** 1 Department of Marine Science, Faculty of Science, Polytechnic University of Marche, Via Brecce Bianche, 60131 Ancona, Italy; 2 Natural History Museum of Denmark, Zoological Museum, Invertebrate Department, Universitetsparken 15, DK-2100 Copenhagen, Denmark

## Abstract

**Background:**

Several unicellular organisms (prokaryotes and protozoa) can live under permanently anoxic conditions. Although a few metazoans can survive temporarily in the absence of oxygen, it is believed that multi-cellular organisms cannot spend their entire life cycle without free oxygen. Deep seas include some of the most extreme ecosystems on Earth, such as the deep hypersaline anoxic basins of the Mediterranean Sea. These are permanently anoxic systems inhabited by a huge and partly unexplored microbial biodiversity.

**Results:**

During the last ten years three oceanographic expeditions were conducted to search for the presence of living fauna in the sediments of the deep anoxic hypersaline L'Atalante basin (Mediterranean Sea). We report here that the sediments of the L'Atalante basin are inhabited by three species of the animal phylum Loricifera (*Spinoloricus *nov. sp., *Rugiloricus *nov. sp. and *Pliciloricus *nov. sp.) new to science. Using radioactive tracers, biochemical analyses, quantitative X-ray microanalysis and infrared spectroscopy, scanning and transmission electron microscopy observations on ultra-sections, we provide evidence that these organisms are metabolically active and show specific adaptations to the extreme conditions of the deep basin, such as the lack of mitochondria, and a large number of hydrogenosome-like organelles, associated with endosymbiotic prokaryotes.

**Conclusions:**

This is the first evidence of a metazoan life cycle that is spent entirely in permanently anoxic sediments. Our findings allow us also to conclude that these metazoans live under anoxic conditions through an obligate anaerobic metabolism that is similar to that demonstrated so far only for unicellular eukaryotes. The discovery of these life forms opens new perspectives for the study of metazoan life in habitats lacking molecular oxygen.

## Background

More than 90% of the ocean biosphere is deep (average depth, 3,850 m) and most of this remains unexplored [1]. The oceans host life at all depths and across the widest ranges of environmental conditions (that is, temperature, salinity, oxygen, pressure), and they represent a huge reservoir of undiscovered biodiversity [2,3]. Deep-sea ecosystems also contain the largest hypoxic and anoxic regions of the Biosphere. The oxygen minimum zones (OMZ) are widely distributed across all of the oceans, at depths generally from 200 m to 1,500 m, and cover approximately 1,150,000 km^2^. These are characterised by very low oxygen availability (O_2 _< 0.5 mM) and high sulphide concentrations in the bottom sediments (>0.1 mM in the surface centimetre) [4]. These environments are inhospitable to most marine species [5], except host prokaryotes, protozoa and some metazoans that can tolerate these environmental conditions [4,6]. Permanently anoxic conditions in the oceans are present in the subsurface seafloor [7], and among other areas, in the interior of the Black Sea (at depths >200 m) [8] and in the deep hypersaline anoxic basins (DHABs) of the Mediterranean Sea [9,10]. All of these extreme environments are assumed to be exclusively inhabited by viruses [11], Bacteria and Archaea [7-10]. The presence of unicellular eukaryotes (for example, protozoan ciliates) in anoxic marine systems has been documented for decades [12] and recent findings have indicated that some benthic foraminifera can be highly adapted to life without oxygen [13]. For limited periods of time, a few metazoan taxa can tolerate anoxic conditions [6,14]. However, so far, there is no proof of the presence of living metazoans that can spend their entire life cycle under permanently anoxic conditions [12].

Metazoan meiofauna (multi-cellular organisms of size ranging from a few micrometres to 1 mm) [15] represent 60% of the metazoa abundance on Earth, and have a long evolutionary history and high phyletic diversity. They include 22 of the 35 animal phyla, six of which are exclusive of the meiofauna (Gnathostomulida, Micrognathozoa, Gastrotricha, Tardigrada, Kinorhyncha, and Loricifera, the most recently described animal phylum) [16]. These phyla lack larval dispersal in the water column and spend their entire life cycle in the sediment. All of these characteristics make meiofauna the ideal organism for investigating metazoan life in systems without oxygen [17,18].

The six DHABs of the Mediterranean Sea are extreme environments at depths >3,000 m that have been created by the flooding of ancient evaporites from the Miocene period (5.5 million years before the present) [19]. Among these, the L'Atalante basin displays a 30 to 60 m thick hypersaline brine layer with a density of 1.23 g cm^-3 ^[9], which represents a physical barrier that hampers oxygen exchange between the anoxic sediments and the surrounding seawaters. This basin is therefore completely oxygen free, rich in hydrogen sulphide, and hosts an incredibly diverse and metabolically active prokaryotic assemblages that have adapted to these conditions [9]. In 1998, 2005 and 2008 we carried out three oceanographic expeditions to search for the presence of living fauna in the sediments of the anoxic L'Atalante basin (Additional file 1).

## Results and Discussion

**Figure 1:**
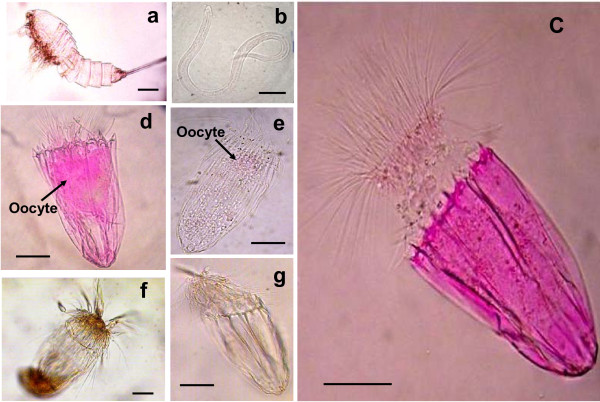
**Metazoans retrieved from the deep hypersaline anoxic L'Atalante basin**. **(a) **Light microscopy (LM) image of a Copepod exuvium (stained with Rose Bengal); **(b) **LM image of dead nematode (stained with Rose Bengal); **(c) **LM image of the undescribed species of *Spinoloricus *(Loricifera; stained with Rose Bengal); **(d) **LM image of the undescribed species of *Spinoloricus *stained with Rose Bengal showing the presence of an oocyte; **(e) **LM image of the undescribed species of *Rugiloricus *(Loricifera, stained with Rose Bengal) with an oocyte; **(f) **LM image of the undescribed species of *Pliciloricus *(Loricifera, non stained with Rose Bengal); **(g) **LM image of moulting exuvium of the undescribed species of *Spinoloricus*. Note the strong staining of the internal structures in the stained loriciferans (c and d) vs. the pale colouration of the copepod and nematode (a, b). The loriciferan illustrated in Figure 1e was repeatedly washed to highlight the presence of the internal oocyte. Scale bars, 50 μm.

In all of the sediments collected from the inner part of the anoxic basin, we found specimens belonging to three animal Phyla: Nematoda, Arthropoda (only Copepoda) and Loricifera. The presence of metazoan meiofauna under permanently anoxic conditions has been reported previously also from the deep-sea sediments of the Black Sea, although these records were interpreted as the result of a *rain of cadavers *that sunk to the anoxic zone from adjacent oxygenated areas [20]. Our specimens collected from the L'Atalante basin were initially stained with a protein-binding stain (Rose Bengal) and examined under the microscope; here, all of the copepods were empty exuviae, and the nematodes were only weakly stained (suggesting that they had been dead for a while, Figure 1a, b), whereas all of the loriciferans, if stained, were intensely coloured (Figure 1c, d). Differences in the colour intensity between live and dead metazoans were confirmed by additional experiments on deep-sea nematodes and copepods (Additional file 2). The taxonomic analysis revealed that the loriciferans collected in the anoxic sediments belong to three species that are new to science and belong to the genera *Spinoloricus *(Figure 1c, similar to the new species of *Spinoloricus turbatio*, which was recently discovered in the deep-sea hydrothermal vents of the Galápagos Spreading Centre) [21], *Rugiloricus *(belonging to the *cauliculus*-group; Figure 1e) and *Pliciloricus *(Figure 1f) [22].

The permanent reducing conditions of anoxic sediments can preserve dead organisms and their protein for a long time, so that microscopic analyses do not provide proof of the viability of an organism. However, the abundance of these loriciferans was the highest reported so far world-wide per unit of surface sediment investigated (range: 75 to 701 individuals m^-2^). This finding is *per se *surprising, as only two individuals of the phylum Loricifera have been found in the deep Mediterranean Sea over the last 40 years [23-25]. Deep-sea oxygenated sediments in the neighboring of the L'Atalante basin were also investigated at the time of sampling as well as in several other occasions since 1989, and we never found one single individual of the phylum Loricifera in the entire Ionian basin. Moreover, the analysis of the oxygenated deep-sea sediments surrounding the L'Atalante basin revealed the dominance of nematodes and copepods (>95% of the total meiofaunal abundance; Additional file 3) and the absence of loriciferans. The density of the Loricifera extracted from the sediment of the L'Atalante basin (determined by density gradient) was 1.15 to 1.18 g cm^-3^, whereas the density of the brines above the sediment is significantly higher (1.23 g cm^-3^). Moreover, the presence of laminated sediment layers along with the lack of turbidites in the L'Atalante basin [26] indicates the lack of lateral transport from adjacent systems. These independent evidences make very unlikely the sedimentation or transfer of Loricifera or their carcasses from the oxygenated sediments surrounding the anoxic basin.

**Figure 2:**
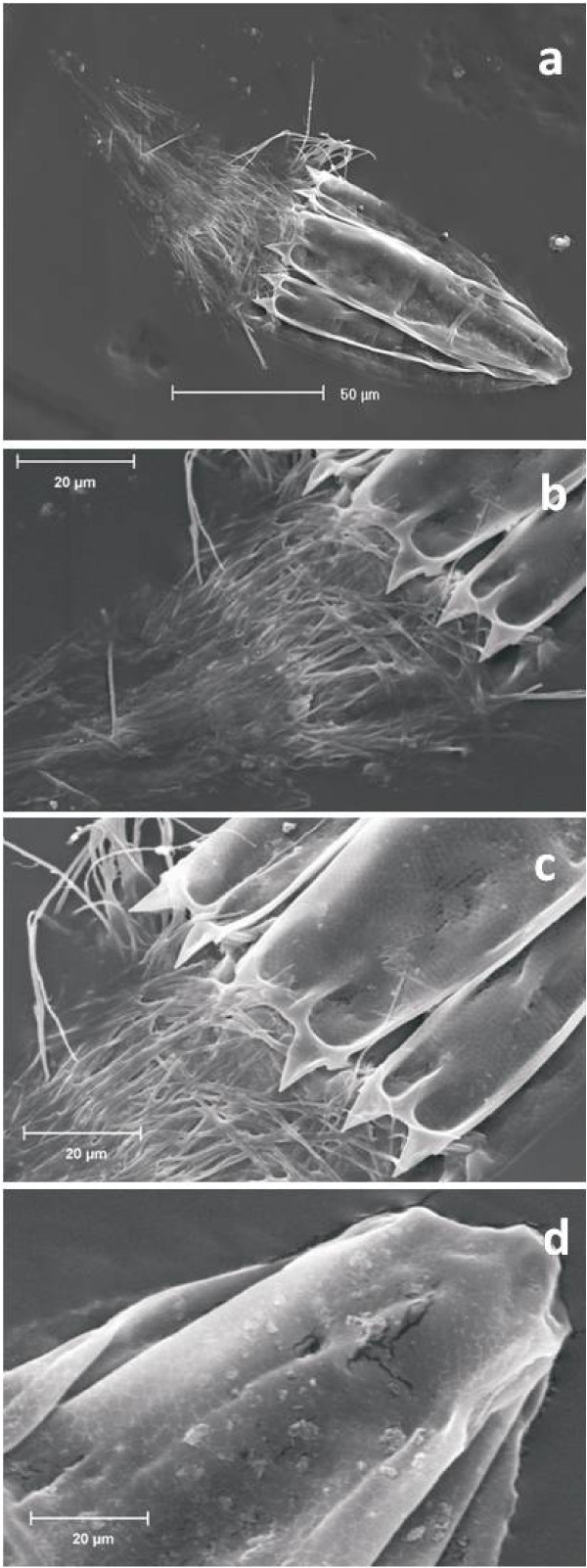
**Morphological details of the undescribed species of *Spinoloricus *(Loricifera)**. Scanning electron microscopy (SEM) image of **(a) **ventral side of a whole animal with the introvert out (note the loricated abdomen with eight plates); **(b-c) **anterior edge of the lorica showing the genus character of the genus *Spinoloricus *(additional spikes); and **(d) **posterior lorica with honey-comb structure. No prokaryotes are evident on the surface of the bodies of the loriciferans. Scale bars, as indicated.

Specimens of the undescribed species of both genera *Spinoloricus *and *Rugiloricus *had a large oocyte in their ovary, which showed a nucleus containing a nucleolus (Figure 1d, e). This is the first evidence of Loricifera reproducing in the entire deep Mediterranean basin. Microscopic analyses also revealed the presence of empty exuviae from moulting loriciferans (Figure 1g), suggesting that these metazoans did grow in this system. Moreover, scanning electron microscopy confirmed the perfect integrity of these loriciferans (Figure 2), while all of the other meiofaunal taxa were largely damaged or degraded.

**Figure 3:**
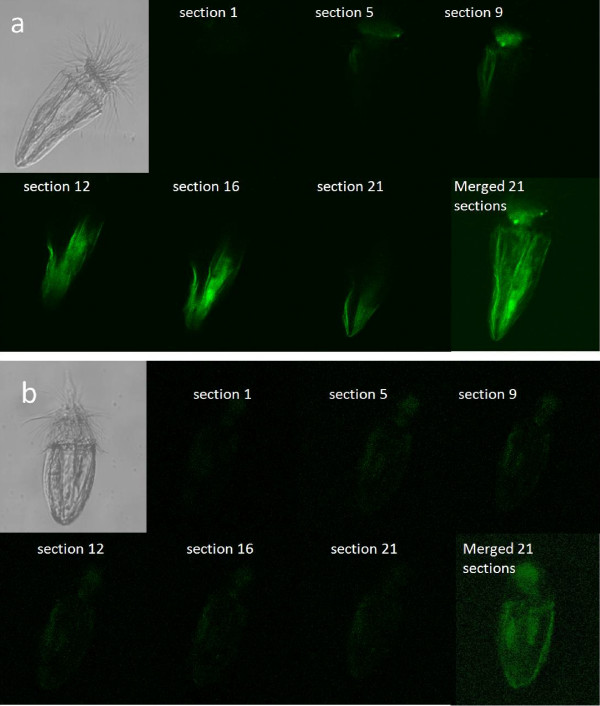
**Incorporation of Cell-Tracker™ Green CMFDA by loriciferans from the anoxic sediments of the L'Atalante basin**. Series of confocal laser microscopy images across different sections of the body volume of the loriciferans. Sections 1-21 represent the progressive scanning of the loriciferans (undescribed species of *Spinoloricus*) from the outer to the inner part of the body. **(a) **Cell-Tracker™ Green CMFDA treated loriciferans; and **(b) **Loriciferans killed by freezing prior to Cell-Tracker™ Green CMFDA treatment and used as a control.

A second expedition was dedicated to the demonstration of the viability of these loriciferans of the L'Atalante basin, through independent experimental approaches. All of the experiments were conducted on deck (101,325 Pa), under anoxic conditions (in a N_2 _atmosphere), in the dark and at the *in-situ *temperature (ca 14°C) immediately after sample retrieval. In the first investigations, intact and undisturbed sediment cores were injected with (^3^H)-leucine (Table 1) to investigate the ability of these loriciferans to take up this radiolabelled amino acid. Following multiple and replicated incubations and controls (that is, loriciferans killed before radiolabelled substrate injection), it was revealed that over a short time scale (four hours), the loriciferans incorporated amounts of this radioactive substrate that were significantly higher than in the controls (that is, killed loriciferans). Decompression can alter significantly metabolic activities of deep-sea organisms during their recovery. However, in our experiments this potential bias was the same for both the controls and the samples containing live Loricifera. Moreover, the ultra-structural analyses did not show any evidence of cell lysis related to the decompression. To test the reliability of the approach utilized we sampled living nematodes from oxygenated sediments and made incubations with (^3^H)-leucine of both living and killed nematodes. Thanks to this experiment we demonstrated the presence of significant differences in the incorporation of radio-labelled compounds and proved the linearity between the number of nematodes and the incorporated radioactivity (Table 1). These results, *per se*, are sufficient to provide compelling evidence of the activity of the organism from the anoxic systems, but we further investigated the viability of the Loricifera collected from the L'Atalante basin by incubating intact and undisturbed sediment cores containing the loriciferans with 5-chloromethylfluorescein diacetate (Cell-Tracker™ Green, CMFDA: Molecular Probes, Inc., Eugene, Oregon, US) which has been previously used to identify living unicellular eukaryotes in anoxic sediments [6]. This fluorogenic probe labels hydrolytically active (that is, live) cells [6]. Comparative analyses conducted on anoxic sediments by confocal laser microscopy on Loricifera kept alive and others that were killed prior to incubation revealed, on average, 40% higher fluorescence intensity in the living Loricifera than in recently killed specimens and the intense fluorescence increased from the outer to the inner parts of the organism (Figure 3a, b). The treatment for the preparation of the controls (that is, Loricifera killed prior to incubation by deep freezing) did not inhibit completely the enzymatic activities present in the body of the animals and therefore we expected the presence of some fluorescence also in the body of the pre-killed animals. This effect has been tested also on different species of living nematodes collected from oxygenated sediments by means of repeated (n = 5) incubation experiments with CellTracker™ Green CMFDA. The differences between living and recently killed nematodes analyzed by confocal laser microscopy were in the same order of the differences encountered between alive and recently killed Loricifera.

**Table 1:** Radiolabelled substrate incorporation in loriciferans from the L'Atalante basin and nematodes from coastal Mediterranean sediments.

	DPM ind^-1^	
	Control (killed)	Treated (living)
**Loriciferans**	53 ± 3	93 ± 4

**Nematodes**		
(n = 1 ind)	56 ± 1	64 ± 3
(n = 2 ind)	58 ± 4	73 ± 7
(n = 5 ind)	61 ± 1	79 ± 4
(n = 10 ind)	62 ± 1	98 ± 2

Reported are individual disintegrations per minute of nematodes (coastal Mediterranean Sea) and loriciferans (L'Atalante basin) incubated with (^3^H)-leucine. Data are means of replicates (n = 3-5) ± standard deviation determined on living specimens (treatment) and Loricifera collected from same anoxic sediments but killed prior to treatment (control). Ind: individuals; DPM: disintegration per minute.

All of these findings provide the first evidence that the anoxic sediments of the L'Atalante basin are colonised by natural populations of loriciferans, and that these metazoans are metabolically active and able to reproduce.

**Figure 4:**
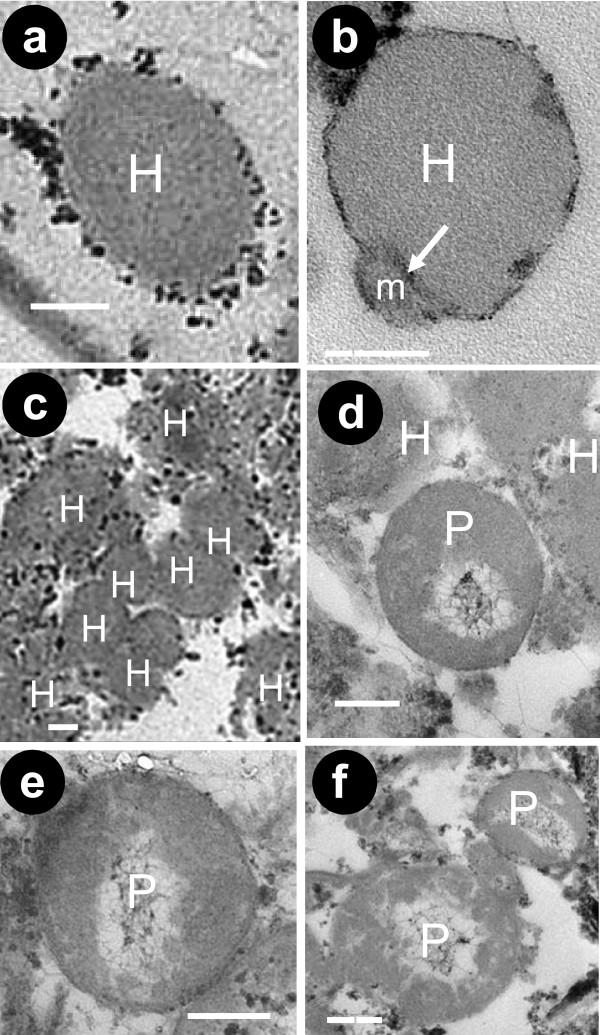
**Electron micrographs of the internal body of loriciferans from the deep hypersaline anoxic L'Atalante basin**. Illustrated are: **(a) **a hydrogenosome-like organelle; **(b) **hydrogenosome-like organelle with evidence of the marginal plate; **(c) **a field of hydrogenosome-like organelles; **(d) **the proximity between a possible endosymbiotic prokaryote and hydrogenosome-like organelles; **(e-f) **the presence of possible endosymbiotic prokaryotes; H = Hydrogenosome-like organelles, P = possible endosymbiotic prokaryotes, m = marginal plate. Scale bars, 0.2 μm.

The adaptations to permanently anoxic conditions associated with high density/salinity and high hydrogen sulphide concentrations imply that these organisms have developed specific mechanisms for: (i) tolerating an enormous osmotic pressure (due to the high salinity and hydrostatic pressure); (ii) detoxifying highly toxic compounds (due to the high hydrogen sulphide concentrations); and (iii) living without oxygen. Quantitative X-ray micro-analysis and Fourier-transformed infrared spectroscopy on the body composition of the loriciferans collected from the anoxic sediments revealed significant differences with the loriciferans collected in the oxygenated deep Atlantic Ocean (Additional files 4, 5 and 6). Loriciferans from the L'Atalante basin had a Ca content (expressed as percentage) that was nine-fold lower than in specimens inhabiting oxygenated sediments, on average, and showed Mg, Br and Fe, which were absent in the loriciferans from oxygenated sediments. Moreover, loriciferans from both oxic and anoxic sediments had similar concentrations of Na and S, in spite of the much higher salinity and sulphide concentration present in the deep-anoxic sediments of the L'Atalante basin (Additional files 4 and 5). Moreover, Fourier-transformed infrared spectroscopy analyses indicated that the lorica of the loriciferans inhabiting oxygenated deep-sea sediments was apparently made of chitin, which was replaced by a chitin derivative, similar to chitosan, in the loriciferans inhabiting anoxic sediments (Additional file 6). These results suggest the presence of chemical/structural adaptations of these loriciferans that can inhabit these anoxic sediments of the L'Atalante basin. Scanning electron microscopy revealed the lack of prokaryotes attached to the body surface of the loriciferans (Figure 2). Ultra-structural analyses carried out by transmission electron microscopy revealed the lack of mitochondria, which are replaced by hydrogenosome-like organelles (Figure 4a, b, c). The hydrogenosome-like structures showed a perfect integrity of their membranes as well as the presence of a marginal plate (Figure 4b). These organelles have been previously encountered in various unrelated unicellular eukaryotes [27,28], but have never been observed so far in multi-cellular organisms (including the facultative anaerobes that face extended periods of aerobiosis during their life cycle) [14]. Moreover, the Loricifera retrieved from anoxic sediments contained hydrogenosome fields (Figure 4c) similar to those reported in anaerobic ciliates [29,30]. Previous studies have reported the ability of multi-cellular organisms to survive in oxygen-free environments, but only for limited periods of time or for a part of their life cycle [14]. The very high abundance of hydrogenosomes within the Loricifera of the L'Atalante basin and the presence of hydrogenosomes fields represent the first discovery for multicellular organisms. Since the hydrogenosomes do not coexist with mitochondria and they are present only in obligate anaerobic eukaryotes (type II anaerobes) [31], these data exclude the possibility that the Loricifera encountered in the anoxic basin are carcasses of organisms inhabiting oxygenated sediments and transported/sedimented into the anoxic basin. Moreover, the transmission electron microscopy also revealed the presence of rod-shaped structures (Figure 4d, e, f), likely prokaryotes, in close proximity to the hydrogenosome-like organelles (Figure 4d). These structures and their spatial distribution resemble the association between hydrogenosomes and methanogenic Archaea, documented so far only in protozoans living in permanently anoxic conditions [29,30].

## Conclusions

The results reported here support the hypothesis that the loriciferans inhabiting the anoxic sediments of the L'Atalante basin have developed an obligate anaerobic metabolism and specific adaptations to live without oxygen. Although the evolutionary/adaptative mechanisms leading to the colonisation of such extreme environments by these metazoans remain an enigma, this discovery opens new perspectives for the study of metazoan life in habitats lacking molecular oxygen.

## Methods

### Study area and sampling

The L'Atalante deep hypersaline anoxic basin (DHAB) was discovered in the Mediterranean Sea in 1993 during an expedition that was a part of the European funded project "Mediterranean Ridge Fluid Flow". The bottom of the L'Atalante basin is a relatively flat area bounded to the southwest by the Cleft Basin and it is characterised by a morphological escarpment that is several hundreds of metres high, which is the sea-bottom expression of the main back thrust of the accretionary ridge. These characteristics originated from the dissolution of buried salt deposits (evaporitic deposits), which remained from the hypersaline waters of the Miocene period (5.5 My before present). The L'Atalante basin is characterised by the presence of a thick brine layer (ca. 40 m) with high density (1.23 g cm^-3^) and high contents of Na^+ ^(4,674 mM), Cl^- ^(5,289 mM) and Mg^+ ^(410 mM) [9]. This layer limits the mixing with the overlying oxic deep-waters to only the upper 1 m to 3 m of the brine, and it additionally acts as a physical barrier for particles settling to the bottom sediments. As a result, the inner part of the L'Atalante basin is completely anoxic since 53,000 yrs before present [32] and is characterized by elevated methane (0.52 mM) and hydrogen sulphide (2.9 mM) concentrations [9]. Undisturbed sediment samples (down to a depth of 30 cm) were collected using a USNEL type box corer (surface ca. 0.2 m^2^), in 1998, 2005, 2006 and 2008. The samples from the DHAB sediment were collected in December 1998 (at 3,363 m depth, 35°18.20'N, 21°23.33'E), August 2005 (at 3,600 m depth, 35°18.23'N, 21°23.33'E), and June 2008 (at 3,450 m depth, 35°18.18'N, 21°23.35'E). In 1998 and 2008 additional sediment samples were collected outside the L'Atalante basin (ca. 10 miles from the DHAB; 35°11.84'N, 21°24.75'E) at ca. 3,250 m depth, for investigation of the characteristics of meiofaunal metazoans from the oxygenated adjacent systems (three sampling sites per period with three to five replicated deployments per site). In the northern-eastern Atlantic Ocean, oxygenated deep-sea sediment samples (55°29.87'N, 15°48.61'W at 600 m depth) were collected during the 2006 expedition. Loriciferans retrieved from these sediments were used for the comparison of their body composition with loriciferan specimens collected in the anoxic sediments of the L'Atalante basin. Sediments retrieved from the deep anoxic basin were immediately processed under strict anaerobic conditions.

### Extraction and identification of benthic metazoans

For the extraction of metazoan fauna from the sediments, the samples (top 15 to 20 cm of the sediment cores) were pre-filtered through a 1,000-μm mesh (to remove larger debris), and a 20-μm mesh was used to retain all of the multi-cellular organisms. The fraction remaining on this latter sieve was re-suspended and centrifuged three times with Ludox HS40 (density 1.31 g cm^-3^) [33]. All of the organisms isolated were counted and classified according to standard protocols [34,35]. Only the organisms collected during the first expedition were stained with Rose Bengal (0.5 g L^-1^), a stain commonly used to highlight the body structures under light microscopy. On average of all collected samples metazoan abundance was 2,075 ind. m^-2 ^in the L'Atalante sediments vs 21,548 ind. m^-2 ^in the oxygenated sediments surrounding the basin. In the anoxic sediments of the L'Atalante basin, Loricifera accounted for 16.1% of the total metazoan abundance. No Loricifera were encountered in the oxygenated sediments surrounding the basin, where nematodes and copepods accounted for 95% and 4%, respectively, of the total metazoan abundance.

### Identification of loriciferans to genus and species level with light and scanning electron microscopy

The extracted specimens were mounted on microslides in a drop of distilled water. The water was progressively replaced by increasing glycerol concentrations (5%, 10%, 25%, 50% and 100% vol water:vol glycerol). Then the specimens were sealed with Glyceel. The microslides were analyzed using a light microscope with phase contrast and Nomarski DIC optics. Micrographs of the specimens were taken on an Olympus BX51 microscope equipped with a digital Olympus C-3030 zoom camera and on a Leica DMRXA microscope with a digital Leica DC200 camera (Leica Camera AG, Solms, Germany). Morphological details of the loriciferans were obtained by scanning electron microscopy. Loriciferans extracted from sediments were carefully rinsed in distilled water and then dehydrated through a graded series of ethanol and acetone prior to critical-point drying. The dried specimens were mounted on aluminium stubs and coated with gold prior to observation under scanning electron microscopy (Philips XL20, Philips Electronics, Eindhoven, The Netherlands).

#### Incubation experiments

##### Incorporation of (^3^H)-leucine

For investigating the vitality of the meiofaunal metazoans, the top 5 cm of intact sediment cores were incubated with (^3^H)-leucine [36]. Replicate sediment samples (n = 3, internal diameter 5.5 cm, approximately 120 cm^3 ^of sediment per replicate sample) were kept in the dark at *in-situ *temperature and under anoxic conditions (a N_2 _atmosphere); these were injected with 10 mL (^3^H)-leucine dissolved in 0.2 μm filtered, autoclaved and degassed deep-sea water (final concentration 0.2 mCi mL^-1^). Controls for the incubation experiments were obtained as follows: additional sediment cores were frozen immediately after collection at -80°C, to kill all metazoans within the samples. After thawing, when the samples reached the *in-situ *temperature, the sediments were incubated with an aqueous solution of (^3^H)-leucine and then processed as described above. We used deep freezing to kill animals, as previous studies have demonstrated that meiofauna fixed using chemical compounds (that is, formaldehyde, glutaraldehyde and ethanol) show a significant loss in the incorporated radioactivity [35]. All samples were incubated on deck (101,325 Pa) under anoxic conditions (N_2 _atmosphere) for four hours in the dark, and at the *in-situ *temperature (about 14°C). At the end of the incubations, the samples were deep-frozen in liquid N_2 _to stop any additional substrate uptake. In the laboratory, the organisms were extracted from the sediment as previously described. Due to the relatively low numbers of loriciferans in the sediment cores (n = 3 both in the control and treated samples) the organisms were analyzed individually. Meiofaunal organisms were rinsed with 0.2-μm pre-filtered seawater (to minimise interference due to radioactivity incorporated by prokaryotes that were potentially present on the metazoan surface) [37] and transferred to scintillation vials. The samples were digested at 50°C for 24 h using 1 mL tissue solubiliser (Soluene-350, Packard Inc., Meriden, Connecticut, US). After addition of 10 mL scintillation cocktail, the radioactivity (as disintegration per minute; DPM) in the loriciferans was determined in a liquid scintillation counter (Packard, Tri-Carb 2100 TR). DPM data were normalised per individual.

To test the accuracy and consistency of the radiotracer experiments carried out on sediments collected in the L'Atalante basin, additional experiments were performed on coastal sediments of the Mediterranean Sea. Loriferans were not present in these samples; therefore nematodes were used as model organisms. After incubation with the radiolabelled substrate, the nematodes (diameter: 20 to 30 μm and length: 200 to 900 μm) were extracted from the sediments and analyzed individually or pooled together (from 2 to 10 individuals). These experiments demonstrated that the radioactivity incorporated into the nematodes is significantly higher than that found in organisms used as controls, even when a single individual is analyzed (Table 1). Moreover, radioactivity measured from the nematodes incubated with radioactive substrates increased linearly with the increasing number of individuals analyzed.

##### Incorporation of Cell-Tracker™ Green CMFDA

After sediment retrieval from the anoxic basin, the top 5 cm of the sediment cores and its anoxic overlying water were maintained under strict anaerobic conditions (N_2 _atmosphere) and incubated on deck (101,325 Pa) in the dark and at the *in-situ *temperature (ca 14°C). The samples were used for incorporation experiments with Cell-Tracker™ Green CMFDA, fluorescent probe (5-chloromethylfluorescein diacetate; Molecular Probes, Inc., Eugene, Oregon, US; 10 μM final concentration). The Cell-Tracker™ Green fluorescent CMFDA probe penetrates the cells and reacts with the intracellular enzymes, generating fluorescence [38]. This molecular probe is specifically designed for testing the presence of metabolic activity and is therefore used here to support the evidence of viability of the metazoans present within the anoxic deep-sea sediments. The sediment samples were incubated for four hours. Controls for the incubation experiments were obtained as follows: additional sediment cores were frozen immediately after collection at -80°C to kill all metazoans within the samples. After thawing, when the samples reached the *in-situ *temperature, the sediments were incubated with an aqueous solution Cell-Tracker™ Green CMFDA, and then processed as described above. At the end of the incubation, the samples were deep-frozen in liquid N_2 _to stop any metabolic reactions, and the recovered loriciferans were placed on concave slides containing a drop of 0.9% NaCl solution (previously autoclaved). The fluorescence of the organisms was examined using a confocal microscope equipped with Kr/Ar mixed gas laser (Bio-Rad MRC 1024 UV; Bio-Rad, Hercules, California, US) using excitation wavelengths 488 nm and the emission has been detected after passing a bandpass filter of 522/35 nm. The confocal laser images were acquired (using the same laser emission power, iris and electronic gain for all acquisitions) in the Bio-Rad *PIC *format using the Bio-Rad Lasersharp Acquisition software (Release 2.1). The organisms were investigated using exactly the same magnification (× 40) in order to allow data comparison. Images were taken at depths of 3 μm for a total of 21 sections per animal and analyzed using the Bio-Rad Lasersharp processing tool. This enabled merging all of the sections (without any contrast manipulation) and measuring the mean scale colour (0 to 255) of the entire animal body. Images were sequentially acquired and stored as TIFF files. The reliability of the control used in the experiment was previously tested by means of repeated (n = 5) incubation experiments with Cell-Tracker™ Green CMFDA performed on two nematode species cultured in the laboratory (*Diplolamelloides myily *and *Diplolaimella diewgatentis*). All of the specimens were analyzed by confocal laser microscopy, as described above.

#### X-ray micro-analysis of the elemental composition of Loricifera

After extraction from the sediment, loriciferans from both the L'Atalante basin (undescribed species of the genus *Spinoloricus*, only adults) and the deep NE Atlantic Ocean (*Rugiloricus cauliculus *cfr) underwent quantitative X-ray micro-analysis, after coating with graphite. Specimens collected in the oxygenated sediments were used as a reference. The selected parts were: abdomen, the posterior lorica and the whole organism (Additional file 4).

#### Spectroscopic infra-red determinations

Fourier transformed Infra-Red (FT-IR) spectroscopic determinations were carried out on loriciferans collected both from the anoxic sediments of the L'Atalante basin and from oxic sediments of the NE Atlantic Ocean. Spectral data were obtained with a Perkin-Elmer Spectrum One FT-IR equipped with a Perkin-Elmer Autoimage microscope (PerkinElmer Life and Analytical Sciences, Shelton, Connecticut, US). Spectra were measured from 4,000 to 400 cm^-1 ^at a spectral resolution of 4 cm^-1 ^with 128 scans. The spatial resolution was 30 × 30 μm. Background scans were obtained from a region of no sample and rationed against the sample spectrum. The samples were deposited first on a steel support to collect reflectance spectra and on the centre of a BaF_2 _plate for transmittance spectral acquisition. Specific areas of interest were identified by means of the microscope television camera. Baseline (polynomial line fit) was performed in all cases while Second Derivative, Fourier Self Deconvolution and Curve Fitting (Gaussian character) procedures were used to determine the absorbance ratio between the bands of interest. All spectra were scaled for equal intensity in the Amide I band. For data handling, the Spectrum v.303 (Perkin-Elmer) software package was used.

#### Analysis of the ultra-structure of loriciferans by transmission electron microscopy

For ultrastructural studies, loriciferans (undescribed species of the genus *Rugiloricus*) extracted from sediments were carefully rinsed in distilled water and then stored in glutaraldehyde (2% final solution) for transmission electron microscopy examinations. After treatment with osmium (one hour incubation) and acetone dehydration (two times at 60% for one minute, and three times at 100% for one minute), loriciferans were embedded in epoxy resin. Ultrathin sections (78 nm) were obtained using a microtome (Model RMC MTX, Boeckeler Instruments Inc., Tucson, Arizona, USA) equipped with a diamond knife. Sections were collected on carbon-coated formvar supports, stained with lead citrate, and examined by transmission electron microscopy (Philips EM 208).

## Abbreviations

OMZ: oxygen minimum zone; DHAB: deep hypersaline anoxic basin; CMFDA: 5-chloromethylfluorescein diacetate (Cell-TrackerTM Green); DPM: disintegration per minute; TIFF: tagged image file format; FT-IR: Fourier transformed infra-red.

## Authors' contributions

RD performed the project planning. AD, CG, KH and RM performed the experimental work, while RD, AD, AP and CG performed the data analysis. RD, AD, AP, CG, KH and RM wrote the manuscript.

## Supplementary Material

Additional file 1 **The study area**. Location of the sampling areas in the central Mediterranean Sea, showing: (a), area (rectangle) including the deep hypersaline anoxic basin (x axis: Longitude; y axis: Latitude); and (b), detailed contour map of the L'Atalante basin. Click here for file

Additional file 2 **The effect of Rose Bengal on living and dead specimens**. (a and b) Light microscopy (LM) images of living deep-sea nematodes collected from oxygenated sediments adjacent to the anoxic basin and stained with Rose Bengal; (c) LM image of dead deep-sea nematode stained with Rose Bengal; (d) LM image of living deep-sea copepods collected from oxygenated sediments adjacent to the anoxic basin and stained with Rose Bengal; (e) LM image of deep-sea copepod exuviae stained with Rose Bengal. Click here for file

Additional file 3 **Meiofaunal abundance and community structure in the L'Atalante basin and the adjacent oxygenated deep-sea sediments**. Comparison of: (a), total abundance of benthic metazoans (expressed as individuals m^-2^) in the anoxic sediments of the L'Atalante deep hypersaline anoxic basin (DHAB) and oxygenated deep-sea sediments surrounding the anoxic basin; and (b), contribution of the different taxa encountered in the anoxic sediments of the L'Atalante DHAB and oxygenated deep-sea sediments surrounding the anoxic basin (expressed as percentages). Click here for file

Additional file 4 **Elemental composition of loriciferans from the L'Atalante basin and oxygenated NE Atlantic deep-sea sediments**. Reported are the relative contents (expressed as percentage) of Na, Mg, Si, P, S, Ca, Fe, Cu, Zn, Br in the abdomen, posterior lorica and whole body of loriciferans. Click here for file

Additional file 5 **Differences in body composition of loriciferans from the L'Atalante basin and the NE Atlantic Ocean**. Output of the principal component analysis carried out on the elemental composition data of different parts of the loriciferans bodies, collected in the L'Atalante basin and in the NE Atlantic Ocean. Vectors in the left part of the panel are proportional to the importance of the investigated chemical elements in distinguishing the Loricifera of the L'Atalante basin from those of the deep-Atlantic oxygenated sediments. Click here for file

Additional file 6 **Fourier-transformed infra-red spectroscopy of loriciferans from the L'Atalante basin and the NE Atlantic Ocean**. Comparison of: (a) Fourier-transformed infra-red spectra of lorificerans collected from the L'Atalante basin (blue line) and from the oxygenated sediments of the NE Atlantic Ocean (red line); (b) spectra of chitosan (black line) and the lorica of the loriciferans collected in the L'Atalante basin (blue line); (c) spectra of chitin (green line) and the lorica of the loriciferans collected from the oxygenated sediments of the NE Atlantic Ocean (red line). Click here for file
