# Supplementary material for: The first metazoa living in permanently anoxic conditions
Source: BMC Biol. 2010 Apr 6;8:30. doi: 10.1186/1741-7007-8-30 (PMC2907586; doi:10.1186/1741-7007-8-30)

Additional File 1: **The study area.** Location of the sampling areas in the central Mediterranean Sea, showing: (a), area (rectangle) including the deep hypersaline anoxic basins (x axis: Longitude; y axis: Latitude); and (b), detailed contour map of the L'Atalante basin.

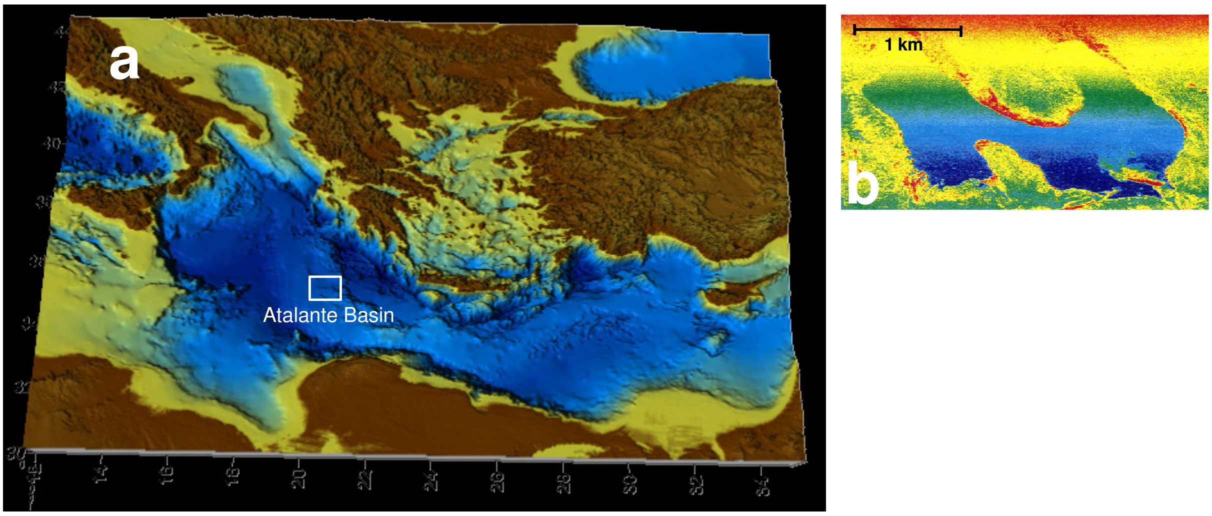

Supplement: Additional file 1 — The study area. Location of the sampling areas in the central Mediterranean Sea, showing: (a), area (rectangle) including the deep hypersaline anoxic basin (x axis: Longitude; y axis: Latitude); and (b), detailed contour map of the L'Atalante basin. [file 1741-7007-8-30-S1.PDF]
