# Supplementary material for: The first metazoa living in permanently anoxic conditions
Source: BMC Biol. 2010 Apr 6;8:30. doi: 10.1186/1741-7007-8-30 (PMC2907586; doi:10.1186/1741-7007-8-30)

Additional File 2: The effect of Rose Bengal on living and dead specimens. (a and b) Light microscopy (LM) images of living deep-sea nematodes collected from oxygenated sediments adjacent to the anoxic basin and stained with Rose Bengal; (c) LM image of dead deep-sea nematode stained with Rose Bengal; (d) LM image of living deep-sea copepods collected from oxygenated sediments adjacent to the anoxic basin and stained with Rose Bengal; (e) LM image of deep-sea copepod exuviae stained with Rose Bengal.

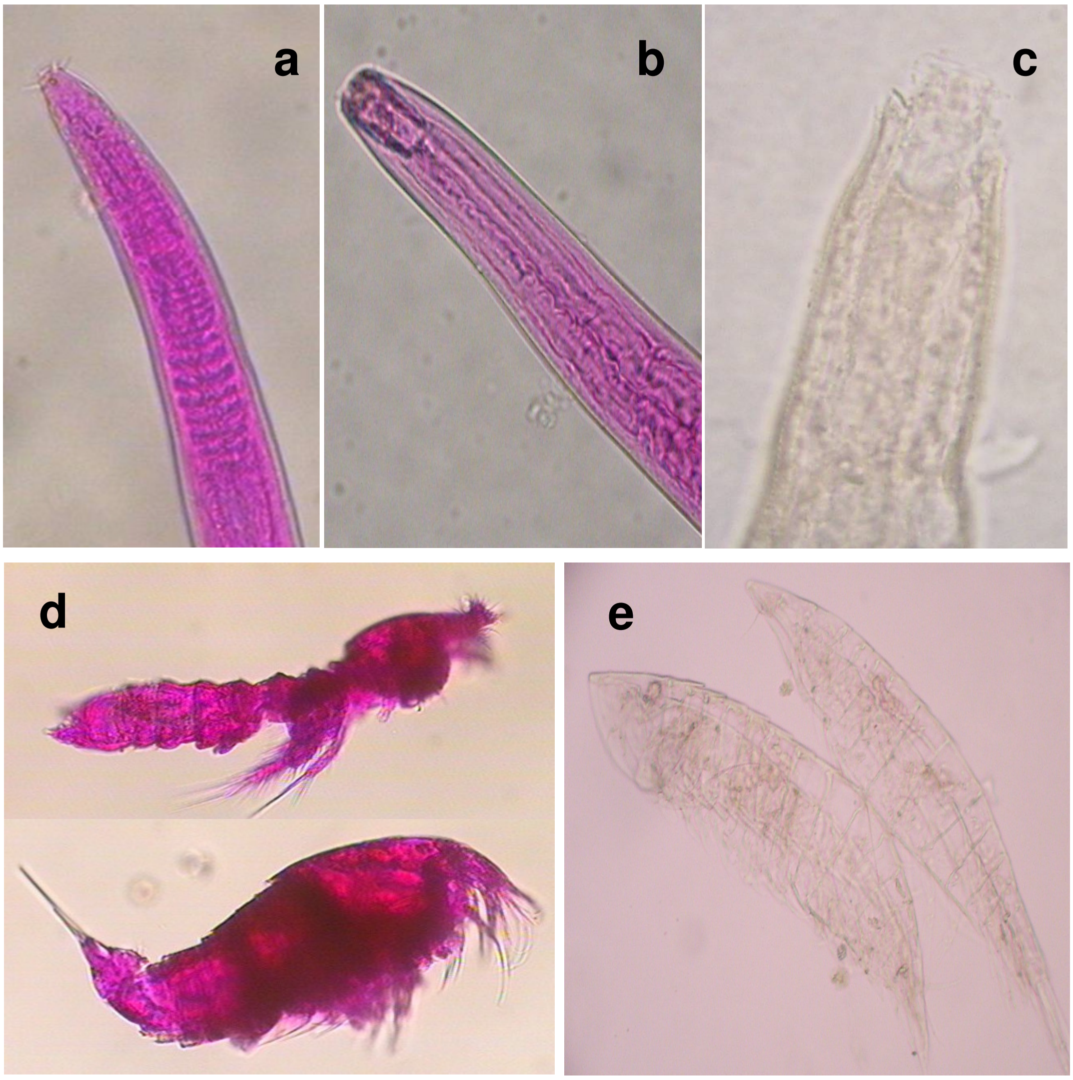

Supplement: Additional file 2 — The effect of Rose Bengal on living and dead specimens. (a and b) Light microscopy (LM) images of living deep-sea nematodes collected from oxygenated sediments adjacent to the anoxic basin and stained with Rose Bengal; (c) LM image of dead deep-sea nematode stained with Rose Bengal; (d) LM image of living deep-sea copepods collected from oxygenated sediments adjacent to the anoxic basin and stained with Rose Bengal; (e) LM image of deep-sea copepod exuviae stained with Rose Bengal. [file 1741-7007-8-30-S2.PDF]
