# Supplementary material for: The first metazoa living in permanently anoxic conditions
Source: BMC Biol. 2010 Apr 6;8:30. doi: 10.1186/1741-7007-8-30 (PMC2907586; doi:10.1186/1741-7007-8-30)

Additional File 3: **Meiofaunal abundance and community structure in the L'Atalante basin and the adjacent oxygenated deep-sea sediments**. Comparison of: (a), total abundance of benthic metazoans (expressed as individuals m^{-2}) in the anoxic sediments of the L'Atalante deep hypersaline anoxic basin (DHAB) and oxygenated deep-sea sediments surrounding the anoxic basin; and (b), contribution of the different taxa encountered in the anoxic sediments of the L'Atalante DHAB and oxygenated deep-sea sediments surrounding the anoxic basin (expressed as percentages).

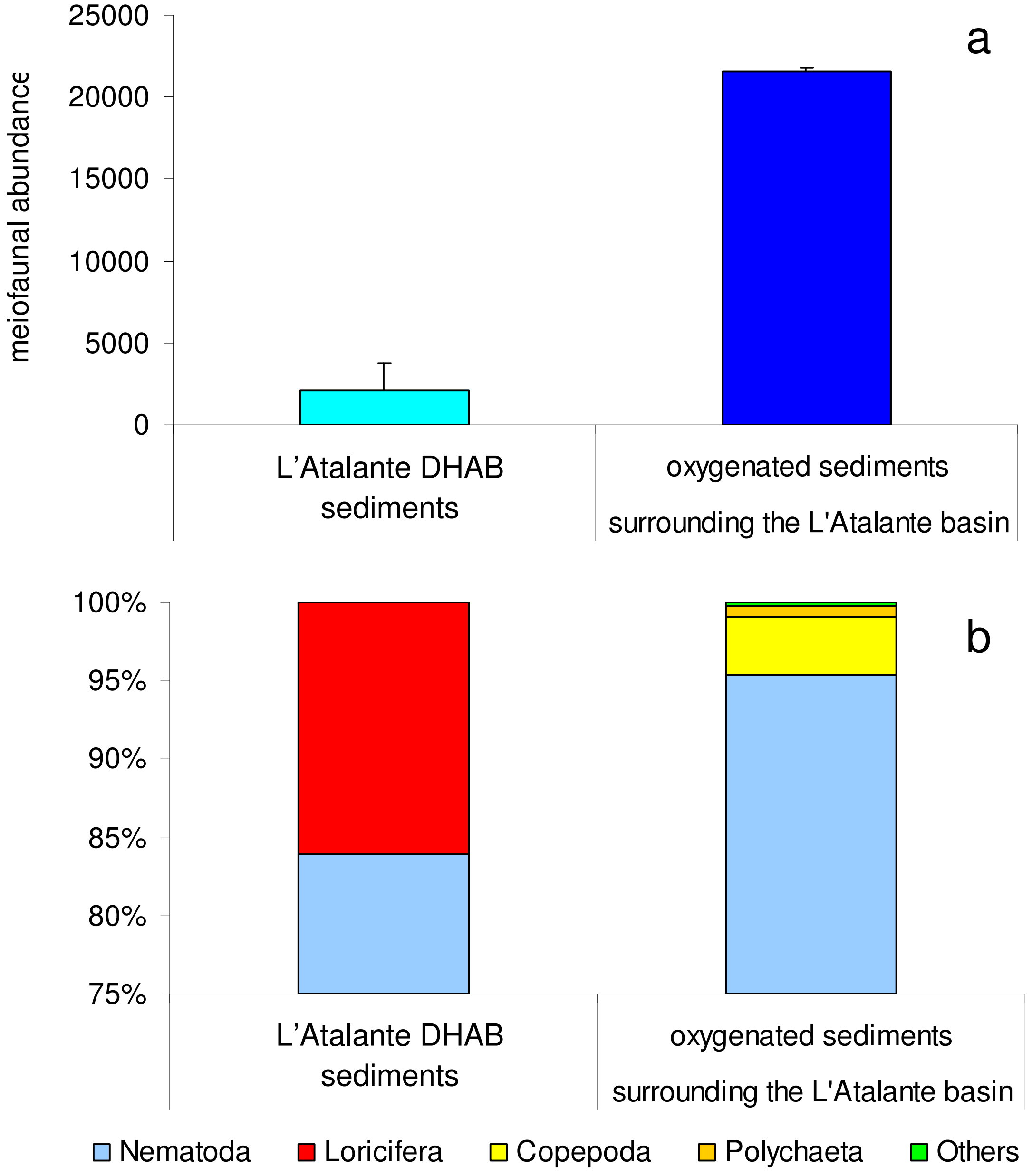

Supplement: Additional file 3 — Meiofaunal abundance and community structure in the L'Atalante basin and the adjacent oxygenated deep-sea sediments. Comparison of: (a), total abundance of benthic metazoans (expressed as individuals m-2) in the anoxic sediments of the L'Atalante deep hypersaline anoxic basin (DHAB) and oxygenated deep-sea sediments surrounding the anoxic basin; and (b), contribution of the different taxa encountered in the anoxic sediments of the L'Atalante DHAB and oxygenated deep-sea sediments surrounding the anoxic basin (expressed as percentages). [file 1741-7007-8-30-S3.PDF]
