# Supplementary material for: The first metazoa living in permanently anoxic conditions
Source: BMC Biol. 2010 Apr 6;8:30. doi: 10.1186/1741-7007-8-30 (PMC2907586; doi:10.1186/1741-7007-8-30)
Supplement: Additional file 4 — Elemental composition of loriciferans from the L'Atalante basin and oxygenated NE Atlantic deep-sea sediments. Reported are the relative contents (expressed as percentage) of Na, Mg, Si, P, S, Ca, Fe, Cu, Zn, Br in the abdomen, posterior lorica and whole body of loriciferans. [file 1741-7007-8-30-S4.PDF]

Additional File 4: Elemental composition of loriciferans from the L'Atalante basin and oxygenated NE Atlantic deep-sea sediments. Reported are the relative contents (expressed as percentage) of Na, Mg, Si, P, S, Ca, Fe, Cu, Zn, Br in the abdomen, posterior lorica and whole body of loriciferans.

**Region**	**Body part**	**Na** %	**Mg** %	**Si** %	**P** %	**S** %	**Ca** %	**Fe** %	**Cu** %	**Zn** %	**Br** %
Atalante basin	Abdomen	0.0	0.0	22.3	9.6	30.6	4.5	0.0	22.5	10.6	0.0
	Posterior	0.0	6.5	31.3	9.9	19.7	2.6	5.2	5.2	1.8	17.7
	Whole	4.6	6.5	21.1	9.1	36.4	2.0	5.2	2.6	1.0	11.5
Atlantic Ocean	Abdomen	15.9	0.0	20.4	8.92	29.5	16.3	0.0	9.22	0.0	0.0
	Posterior	0.0	0.0	4.2	17.1	29.4	33.1	0.0	7.2	9.0	0.0
	Whole	4.94	0.0	10.7	12.3	31.0	28.4	0.0	5.9	6.8	0.0
