# Supplementary material for: The first metazoa living in permanently anoxic conditions
Source: BMC Biol. 2010 Apr 6;8:30. doi: 10.1186/1741-7007-8-30 (PMC2907586; doi:10.1186/1741-7007-8-30)

Additional File 5: Differences in body composition of loriciferans from the L'Atalante basin and the NE Atlantic Ocean. Output of the principal component analysis carried out on the elemental composition data of different parts of the loriciferans bodies, collected in the L'Atalante basin and in the NE Atlantic Ocean. Vectors in the left part of the panel are proportional to the importance of the investigated chemical elements in distinguishing the Loricifera of the L'Atalante basin from those of the deep-Atlantic oxygenated sediments.

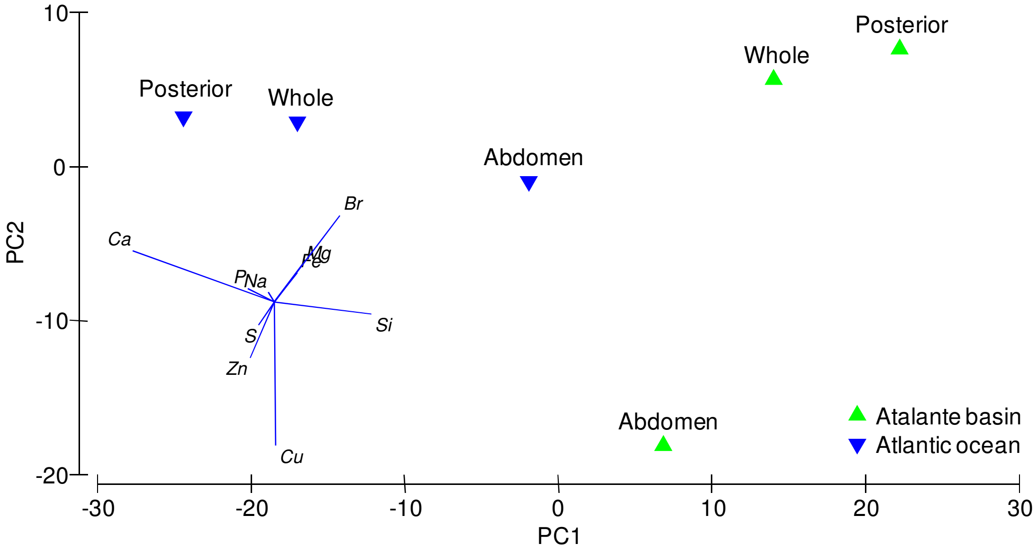

Supplement: Additional file 5 — Differences in body composition of loriciferans from the L'Atalante basin and the NE Atlantic Ocean. Output of the principal component analysis carried out on the elemental composition data of different parts of the loriciferans bodies, collected in the L'Atalante basin and in the NE Atlantic Ocean. Vectors in the left part of the panel are proportional to the importance of the investigated chemical elements in distinguishing the Loricifera of the L'Atalante basin from those of the deep-Atlantic oxygenated sediments. [file 1741-7007-8-30-S5.PDF]
