# Supplementary material for: The first metazoa living in permanently anoxic conditions
Source: BMC Biol. 2010 Apr 6;8:30. doi: 10.1186/1741-7007-8-30 (PMC2907586; doi:10.1186/1741-7007-8-30)

Additional File 6: Fourier-transformed infra-red spectroscopy of loriciferans from the L'Atalante basin and the NE Atlantic Ocean. Comparison of: (a) Fourier-transformed infra-red spectra of lorificerans collected from the L'Atalante basin (blue line) and from the oxygenated sediments of the NE Atlantic Ocean (red line); (b) spectra of chitosan (black line) and the lorica of the loriciferans collected in the L'Atalante basin (blue line); (c) spectra of chitin (green line) and the lorica of the loriciferans collected from the oxygenated sediments of the NE Atlantic Ocean (red line).

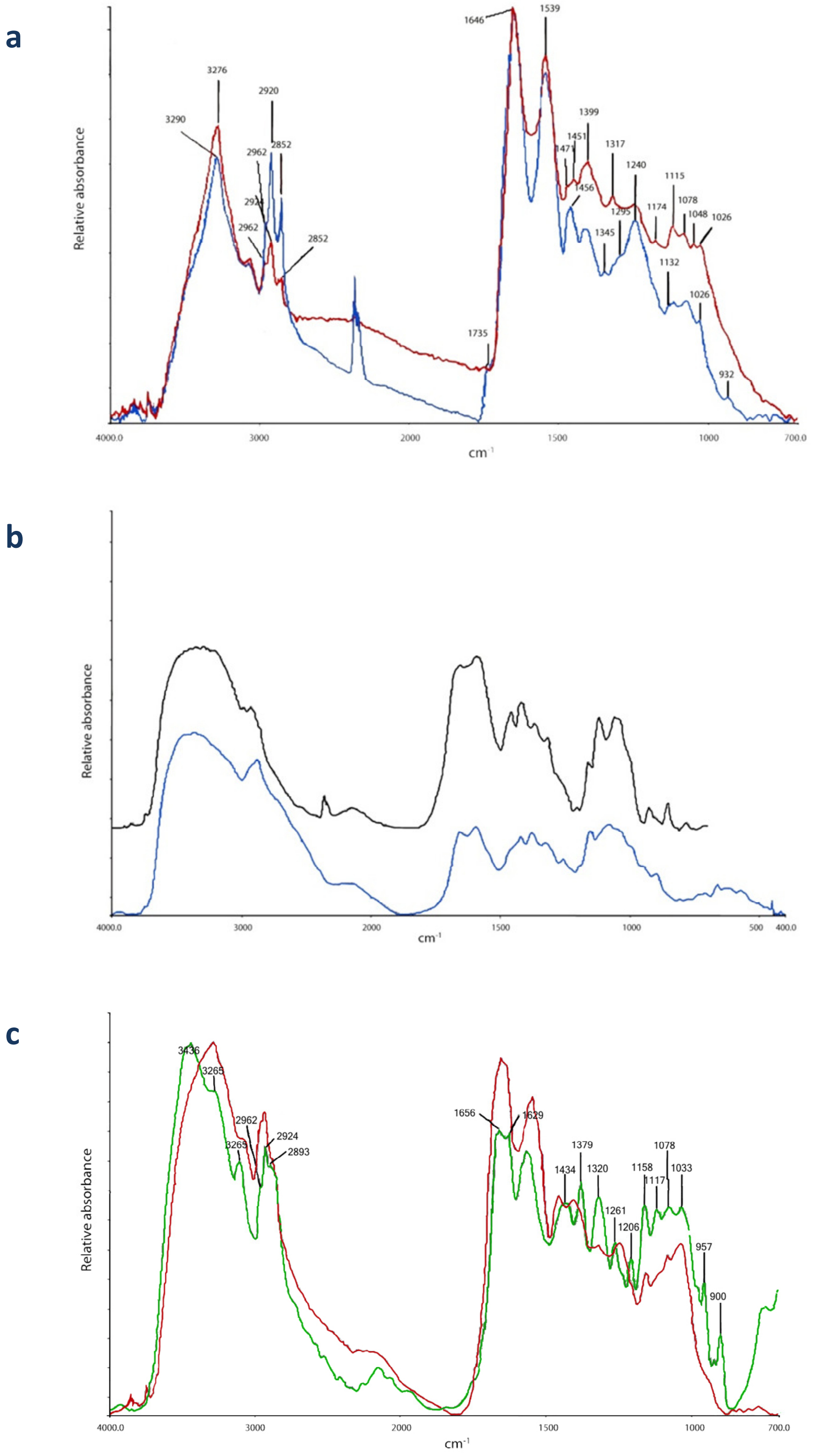

Supplement: Additional file 6 — Fourier-transformed infra-red spectroscopy of loriciferans from the L'Atalante basin and the NE Atlantic Ocean. Comparison of: (a) Fourier-transformed infra-red spectra of lorificerans collected from the L'Atalante basin (blue line) and from the oxygenated sediments of the NE Atlantic Ocean (red line); (b) spectra of chitosan (black line) and the lorica of the loriciferans collected in the L'Atalante basin (blue line); (c) spectra of chitin (green line) and the lorica of the loriciferans collected from the oxygenated sediments of the NE Atlantic Ocean (red line). [file 1741-7007-8-30-S6.PDF]
